# An Unexpected Diagnosis of Kawasaki Disease in a Three-Month-Old Infant: A Diagnostic Trap

**DOI:** 10.7759/cureus.87088

**Published:** 2025-07-01

**Authors:** Olfa Asbik, Ikram El Hachmi, Aziza Elouali, Maria Rkain, Abdeladim Babakhouya

**Affiliations:** 1 Department of Pediatrics, Mohammed VI University Hospital, Faculty of Medicine and Pharmacy of Oujda, Mohammed I University, Oujda, MAR

**Keywords:** complications, coronary aneurysm, fever, kawasaki, young infant

## Abstract

Kawasaki disease (KD) is an acute systemic vasculitis predominantly affecting young children and can lead to serious cardiac complications if not diagnosed and treated promptly. Diagnosing KD in infants younger than six months is challenging due to atypical or incomplete presentations, increasing the risk of cardiovascular complications. The purpose of this case report was to detail the clinical presentation, laboratory findings, and cardiac outcomes in a patient diagnosed with KD during the first three months of life. The infant’s initial symptoms were misleading, as the prolonged fever was initially attributed to pyelonephritis, leading to an antibiotic treatment that did not resolve the fever. The overlap of KD symptoms with other febrile illnesses and the subtle or absent classical signs of KD in this age group often complicate the diagnosis. This case underscores the importance of considering KD in infants under six months with persistent fever and highlights diagnostic challenges, including misinterpretation of symptoms. Early echocardiography and high clinical suspicion are crucial for timely diagnosis and management to prevent severe cardiac complications in this vulnerable age group.

## Introduction

Kawasaki disease (KD) is an acute systemic vasculitis of unknown cause that primarily affects children between six months and five years of age [1]. It occurs worldwide and is the leading cause of acquired heart disease in children in developed nations. Since there are no pathognomonic tests, diagnosis depends on recognizing key clinical features and excluding other diseases with similar presentations [1]. Diagnosing KD in very young infants is particularly difficult; it should be suspected in any child aged three months or younger who has a persistent unexplained high fever, even if they exhibit only some or none of the typical KD symptoms [2]. Additionally, these younger patients face a greater risk of cardiovascular complications, atypical or incomplete clinical signs, and Kawasaki Disease Shock Syndrome (KDSS), necessitating heightened clinical vigilance [2]. The cause of KD remains unknown, but it is hypothesized to involve an abnormal immune reaction triggered by infectious agents in genetically susceptible individuals. 

We present the case of a three-month-old infant diagnosed with KD, whose diagnosis was challenging and initially confused with pyelonephritis. This report details the clinical features, diagnostic approach, and treatment strategies employed.

## Case presentation

A three-month-old infant, born to parents in a first-degree consanguineous marriage, was delivered at term following a well-monitored pregnancy without significant complications. The vaginal delivery was medically assisted. The infant had a birth weight of 3.5 kg and was breastfed.

Initially admitted to our department at the age of two months for the management of isolated acute fever, a diagnosis of pyelonephritis was made based on leukocyturia of 80,000/ml, which was treated with a dual antibiotic regimen. On the fifth day of hospitalization, he developed a maculopapular rash, attributed to an allergic reaction, and subsequently managed with corticosteroids, resulting in a favorable recovery. After a 10-day hospital stay, the infant was discharged upon achieving apyrexia. However, the progression was marked by the recurrence of fever three days after discharge, which prompted a consultation with an otolaryngologist, who diagnosed otitis media. He was restarted on antibiotics, but without improvement. The fever continued for an additional 20 days, leading to her admission to our department for further investigation.

Clinical examination upon admission revealed an irritable infant, hemodynamically and respiratorily stable, with a fever of 38.5°C and no signs of dehydration. Examination of the skin and mucous membranes revealed cheilitis, edema of the extremities (hands and feet), and periungual desquamation. Laboratory results are shown in Table 1.

**Table 1:** Laboratory Findings and Reference Ranges of the Patient

Lab Parameters	Patient's Normal Values	Reference Range
Platelets	606,000 / µl	150 000,00 - 400 000,00/µl
Leukocytosis	27000 / µl	4 000,00 - 10 000,00 /µl
Neutrophils (PNN)	15860 / µl	1 500,00 - 7 000,00) /µl
Lymphocytes	6400/ µl	1 000,00 - 4 000,00 /µl
hemoglobin	11.8	12 - 16g/dl
C-reactive protein (CRP)	196 mg/l	< 5 mg/L
erythrocyte sedimentation rate (ESR)	110 mm/h	00.00-20 mm/h
Albumin	30g/l	35 - 50 g/l
Pyuria	150000 ml	<10000 ml

**Figure 1:**
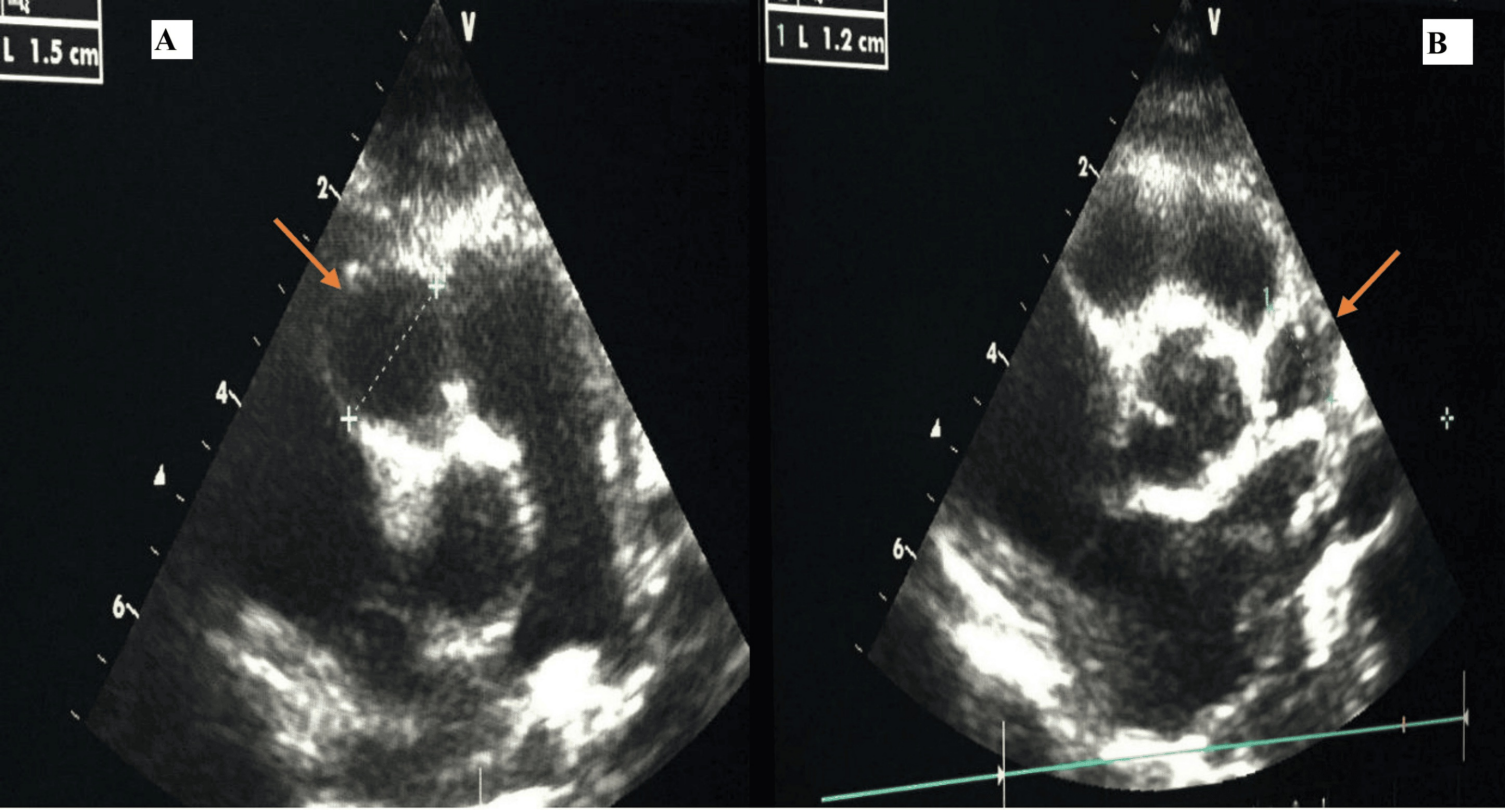
Echocardiogram Revealing Fusiform Aneurysms in Coronary Arteries: 15 mm in the Right and 12 mm in the Left

Given the clinical findings, a diagnosis of KD was suspected, prompting the performance of an echocardiogram. The echocardiogram revealed two fusiform aneurysms in the coronary arteries: one measuring 15 mm in the right coronary artery and another measuring 12 mm in the left coronary artery (see Figure 1).

The infant was promptly treated with intravenous immunoglobulin at a dose of 2 g/kg over 48 hours, in conjunction with acetylsalicylic acid (aspirin) at 100 mg/kg/day and anticoagulation therapy with a vitamin K antagonist; however, due to significant hemorrhagic episodes, it was necessary to discontinue this therapy (vitamin K antagonists), and a switch to clopidogrel was initiated. The clinical course was favorable, with significant improvement in the infant's condition, and he responded well to the treatment regimen. Regular follow-up was implemented, initially every three months and later extended to every six months. Currently, the patient is in a stable condition. The aneurysm on the left side measures 6 mm, while the one on the right side measures 5 mm.

## Discussion

According to the 23rd National Census of Kawasaki disease in Japan, Makino et al. reported that the majority of cases occurred between nine and 11 months of age [3]. Similarly, the most recent Korean survey identified 29 months as the most common age of onset for KD [4]. In Mexico, national data collected from 2000 to 2018 indicated that the typical age at diagnosis was 12 months [2].

KD is rare in very young infants. The highest incidence is typically observed between six months and two years of age. Nonetheless, cases occurring in infants under six months are less frequent, making diagnosis more challenging. The largest investigation focusing on younger patients was carried out in Korea by Lee et al., using data from national surveys. Out of 27,851 diagnosed cases, only 609 (2.18%) involved children younger than three months [5]. In a Mexican cohort, Garrido-García et al. analyzed 688 KD cases from 1995 to 2019, finding that 15 cases (2.3%) were in infants under six months [2].

Patients with KD who are younger than six months often exhibit incomplete clinical symptoms, which can delay diagnosis and lead to a higher incidence of cardiac complications compared to older children. Salgado et al. reported coronary lesions in 43.4% of infants younger than six months, compared to 19.5% in those older than six months [6]. Similarly, Singh et al. documented a 35% incidence of coronary lesions in patients under six months of age [7].

Ram Krishna et al. investigated predictors of coronary artery aneurysms and found that anemia, low albumin levels, elevated erythrocyte sedimentation rate, increased C-reactive protein, and pyuria are significant risk factors for coronary artery abnormalities [8]. Similarly, Bayers et al. reported associations between coronary artery lesions and elevated neutrophil counts, high ESR, low albumin, and decreased hemoglobin levels [9]. These findings suggest a more severe inflammatory response in KD patients aged ≤6 months. This clinical presentation was observed in our patient [10].

A key diagnostic pitfall in this case was the misinterpretation of leukocyturia as indicative of a urinary tract infection, leading to unnecessary antibiotic treatment. Additionally, the maculopapular rash was erroneously attributed to an allergic reaction rather than being recognized as a potential systemic manifestation of an underlying inflammatory condition. These misjudgments resulted in a delayed diagnosis, postponing appropriate management and potentially affecting disease progression. This makes the diagnosis particularly challenging in this age group.

## Conclusions

In conclusion, infants under six months of age presenting with an unexplained fever persisting for more than five days warrant a high index of suspicion for KD, even in the absence of the full spectrum of classical clinical manifestations. Early recognition of this condition is crucial, as delayed diagnosis can lead to serious cardiovascular complications, including coronary artery aneurysms. Therefore, clinicians should maintain vigilance and consider KD in their differential diagnosis when faced with prolonged fever in this age group. Echocardiography serves as an essential diagnostic tool and should be employed promptly and judiciously to evaluate suspected cases, enabling timely intervention and improved patient outcomes.
